# Chronic constrictive pericarditis with rheumatic mitral regurgitation

**DOI:** 10.1093/jscr/rjae424

**Published:** 2025-02-10

**Authors:** Mohammed Tribak, Christ-Marion Adanho, Yassine Elmourabit, Lalla Hasna Leghlimi, Aida Soufiani, Zineb Agoumy, Samah El-Mhadi, Lahcen Marmade

**Affiliations:** Department of Cardiovascular Surgery “B”, Ibn Sina University Hospital, Mohamed V University, Rabat 10800, Morocco; Department of Cardiovascular Surgery “B”, Ibn Sina University Hospital, Mohamed V University, Rabat 10800, Morocco; Department of Cardiovascular Surgery “B”, Ibn Sina University Hospital, Mohamed V University, Rabat 10800, Morocco; Department of Cardiovascular Anesthesia “B”, Ibn Sina University Hospital, Mohamed V University, Rabat 10800, Morocco; Department of Cardiology “A”, Ibn Sina University Hospital, Mohamed V University, Rabat 10800, Morocco; Department of Cardiology “A”, Ibn Sina University Hospital, Mohamed V University, Rabat 10800, Morocco; Department of Cardiology “A”, Ibn Sina University Hospital, Mohamed V University, Rabat 10800, Morocco; Department of Cardiovascular Surgery “B”, Ibn Sina University Hospital, Mohamed V University, Rabat 10800, Morocco

**Keywords:** chronic constrictive pericarditis, mitral regurgitation, pericardectomy, rheumatic

## Abstract

The case of a 31-year-old woman presenting with chronic constrictive pericarditis and mitral valve regurgitation secondary to rheumatic heart disease is described along with a review of the literature with the aim to highlight this uncommon association.

## Introduction

Constrictive pericarditis is a chronic inflammatory process that leads to pericardial thickening and compression of the heart. The cause is idiopathic in the majority of cases, and in some cases, it includes acute pericarditis, infection, malignancy, radiation, rheumatoid disease, trauma, and previous cardiotomy [1]. Pericardiectomy is the treatment of choice, which usually results in immediate relief of constriction and subsequently in improved hemodynamics without adverse sequelae [1, 2]. Association of rheumatic mitral regurgitation and chronic constrictive pericarditis is rare and very few cases have been described in the literature [3, 4]. Here we report an unusual case of severe mitral regurgitation associated with chronic constrictive pericarditis.

## Case presentation

A 31-year-old woman was admitted to our hospital for history of ascites that had been ongoing for 11 years. Past medical history was marked by paracentesis revealed a transudate fluid with no evidence of tuberculosis or systemic disease. She had also a moderate mitral insufficiency. One year ago, the severity of the symptoms had worsened with persistence of ascites and shortness of breath.

At admission, patient was in good general health, conscious, eupneic in rest. Heart rate was 120 beats/min, blood pressure was 100/50 mmHg and temperature was 37°C. Physical examination showed congestive right heart failure, including jugular venous distension, ascites and retromalleolar edema.

Biological examinations showed a hemoglobin level at 10.3 g/100 ml, white blood cells at 6200/mm^3^, platelet count at 199 000/mm^3^, prothrombin level at 32%, urea at 0.19 g/L, creatinine at 9.4 mg/L, and C reactive protein at 26 mg/L.

Chest auscultation revealed an irregular cardiac rhythm with holosystolic murmur at the apex and sytolic murmur at the xyphoid appendix that increases with inspiration in relation with respectively a mitral and tricuspid regurgitation.

**Figure 1:**
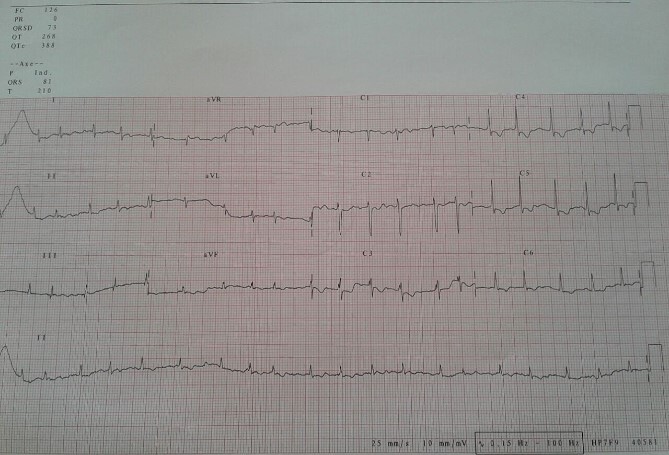
Electrocardiogram showed atrial fibrillation tachycardia.

**Figure 2:**
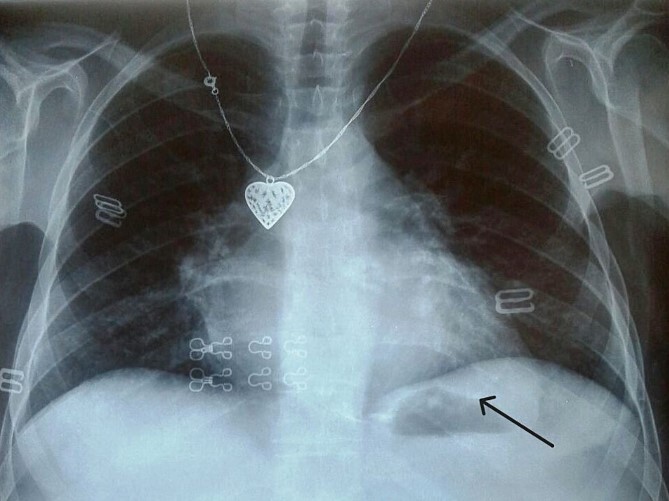
Chest X ray showed pericardial calcification around heart (arrow).

**Figure 3:**
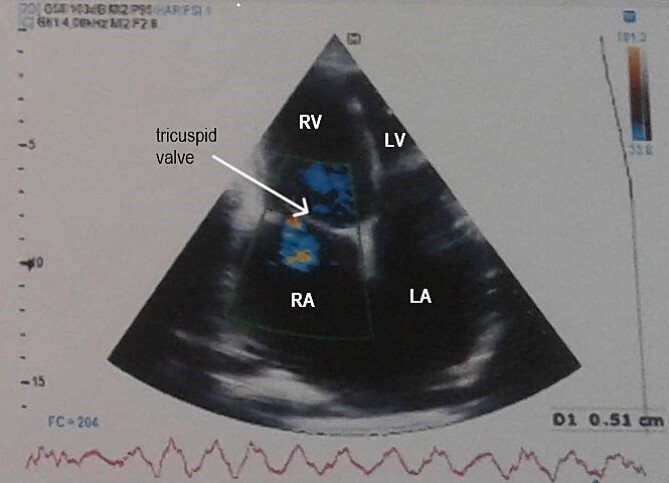
Transthoracic echocardiography showed pericardial thickening, moderate tricuspid regurgitation and biatrial dilation. RA: right atrium, RV: right ventricle, LA: left atrium, LV: left ventricle.

**Figure 4:**
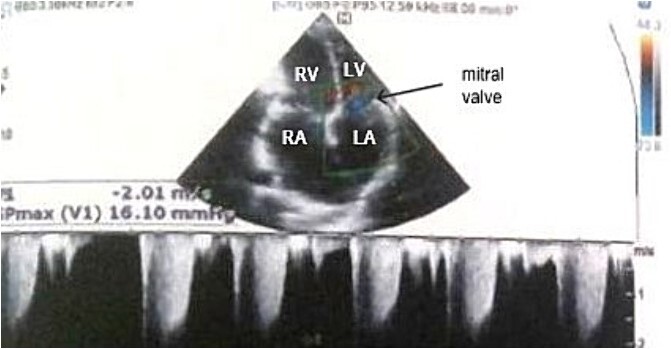
Transthoracic echocardiography showed moderate mitral regurgitation. RA: right atrium, RV: right ventricle, LA: left atrium, LV: left ventricle.

**Figure 5:**
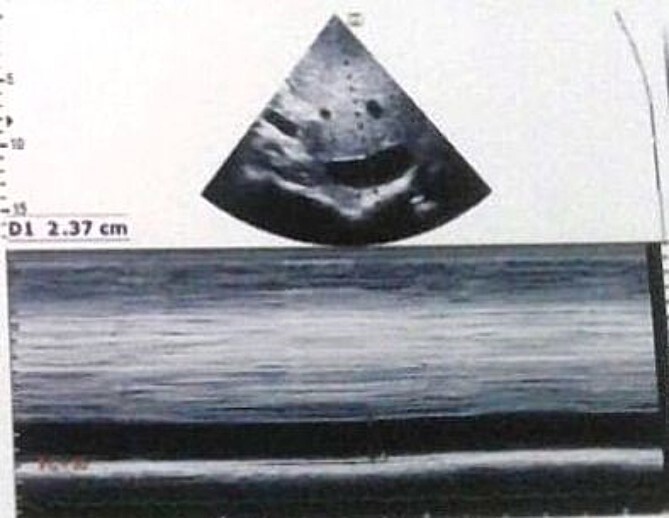
Transthoracic echocardiography showed inferior vena cava dilation.

Electrocardiogram (Fig. 1) showed an atrial fibrillation at 120 c/mn with normal axis and a T wave inversion in anterior. Chest radiography (Fig. 2) revealed pericardial calcifications and a mild cardiomegaly. Transthoracic echocardiography (Figs 3–5) showed a pericardial thickening, an important distension of left atrium (LA diameter = 66 mm, LA surface = 33 cm^2^) and the inferior vena cava (23.7 mm), a calcified mitral leaflets as well as the subvalvular apparatus, restriction of the posterior mitral leaflet with moderate mitral regurgitation (Pisa: 6 mm and ERO: 35 cm^2^), dilated right heart cavities with moderate tricuspid regurgitation and severe pulmonary hypertension (62 mmHg), preserved left ventricular systolic function (EF LV: 63%) with paradoxical septum. The patient was on lasilix 40 mg one tablet per day, aldactone 50 mg one tablet per day and digoxin 0.25 mg one tablet per day.

**Figure 6:**
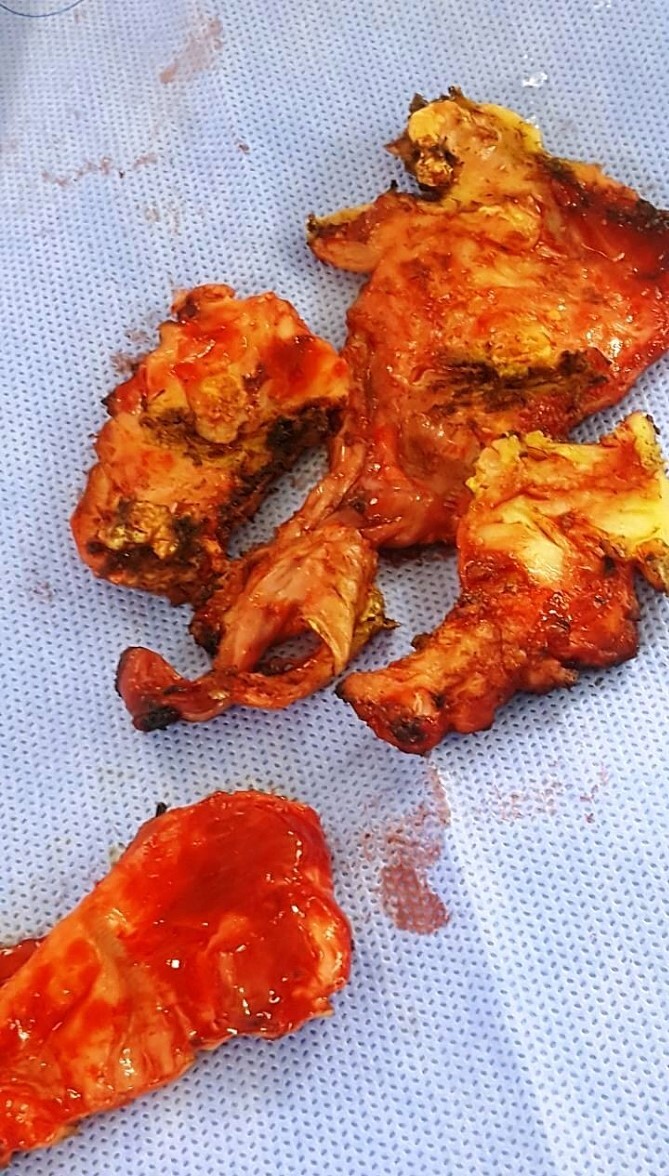
Histological specimen of pericardial tissue.

Because the findings were thought to be most compatible with constrictive pericarditis and rheumatic mitral valve disease, a subtotal pericardiectomy and mitral valve surgery were performed. Peroperative finding (Fig. 6) shows a thickened and calcified pericarditis with an extensive calcifications embedded in the myocardium on the anterior and inferior side of the right ventricle. She underwent ‘phrenic-to-phrenic’ pericardiectomy. The macroscopic appearance finds dilated right heart cavities and a slightly dilated pulmonary artery.

On the mitral side, a dilated left atrium was found, both mitral valve leaflets and subvalvular apparatus were calcified, thickened and shortened. Mitral annulus was dilated. Mitral valve was replaced using a mechanical prosthesis (Mechanical Heart Valve; size 29; Saint Jude medical Masters series) with partial preservation of the posterior valvular apparatus. On the tricuspid side, the leaflets were thickened in the free edges with retraction of the septal valve and the tricuspid annulus was dilated. The tricuspid valve was repaired using an annuloplasty ring (Profile 3D Tricuspid Annuloplasty Ring; size 30; Medtronic).

Postoperatively, she remained in the intensive care unit with low doses of positive inotropes. At the sixth postoperative hours, she experienced significant bleeding. Transthoracic echocardiography revealed a pericardial effusion and compressive hematoma around the right atrium which required surgical hemostasis. Microscopical examination of the excised pericardium failed to demonstrate a cause. Patient was discharged after 10 days. A total of 1-, 3- and 6-months cardiologist check-ups found the patient in good clinical condition without symptoms of heart failure. Transthoracic echocardiography showed preserved left ventricular function, normal prosthetic mitral valve function without tricuspid regurgitation.

## Discussion

The particularity of our case is the association of a rheumatic mitral regurgitation and a chronic constrictive pericarditis. This association is rare and his diagnosis and treatment management is difficult.

Clinical symptomatology of chronic constrictive pericarditis is predominant over symptoms of mitral regurgitation which is attenuate. This is due to pericardial compression limiting expansion of mitral annulus with attenuation of mitral regurgitation. This mechanism is relatively similar to that reported by Srichai *et al*. [5] with acute mitral regurgitation secondary to papillary muscle rupture masked by the presence of cardiac tamponade.

According to Lin [6], diastolic dysfunction is present because of pericardial constriction, the heart cannot expand completely, and a smaller left ventricular end-diastolic diameter indicates more severe pericardial constriction.

Existence of rheumatic mitral regurgitation at baseline is an aggravating factor and it may be progressive. However, functional mitral regurgitation may exist in patients with chronic constrictive pericarditis. Clinical expression of mitral regurgitation will be masked or attenuated by chronic constrictive pericarditis initially. Over time, worsening mitral regurgitation and symptoms of mitral regurgitation is added to that of the chronic constrictive pericarditis, this was the case in our patient.

Uchikawa *et al.* postulate that potential mechanisms of worsening mitral regurgitation in a patient with chronic constrictive pericarditis are due to tight pericardium adhesion to the myocardium, which could increase tension over the mitral apparatus, induce a change in the left ventricular geometry, impaired mitral valve function, and cause myxomatous mitral valve disease through mechanical stress or chronic inflammation [7].

The chronic constrictive pericarditis, over time, damages the heart muscle with impaired hemodynamic making appear or worsen mitral regurgitation [8].

Mantri and al reported in six out of seven patients in whom regurgitations did not regress after pericardectomy, persistent hemodynamic abnormality suggesting indirectly that these regurgitations are in some way related to the abnormal hemodynamics of pericardial constriction [8].

Management of this combination is a subtotal pericardectomy associated with mitral valve surgery. In our patient, we opted for mitral valve replacement in the presence of rheumatic lesions affecting the mitral leaflets and the subvalvular apparatus.

## Conclusion

This case is unusual in that two pathologies, chronic constrictive pericarditis and rheumatic mitral regurgitation are associated with intricate hemodynamic consequences. This illustrates the impact of chronic constrictive pericarditis on the left ventricular repercussion of mitral regurgitation by symptoms attenuation. Surgical management includes subtotal pericardectomy associated with mitral valve repair or replacement.

## Acknowledgements

The authors would like to thank Dr Omar Ech-cherif Elkettani, Dr Youssef Saadouni, Pr Nesma Bendagha, Dr Fadoua Lachhab and Pr Said Moughil for their contribution to draft the manuscript.
